# *Pisidium coreanum* Inhibits Multinucleated Osteoclast Formation and Prevents Estrogen-Deficient Osteoporosis

**DOI:** 10.3390/ijms20236076

**Published:** 2019-12-02

**Authors:** Mun Hwan Choi, Kyunghee Lee, Mi Yeong Kim, Hong-In Shin, Daewon Jeong

**Affiliations:** 1 Department of Microbiology, Laboratory of Bone Metabolism and Control, Yeungnam University College of Medicine, Daegu 42415, Korea; choibak@ynu.ac.kr (M.H.C.); kyungheelee@ynu.ac.kr (K.L.); gsungmi1004@naver.com (M.Y.K.); 2 Department of Oral Pathology, Institute for Hard Tissue and Bio-Tooth Regeneration, School of Dentistry, Kyungpook National University, Daegu 41940, Korea; hishin@knu.ac.kr

**Keywords:** *Pisidium coreanum*, bone, osteoporosis, trabecular bone, cortical bone, osteoclast fusion

## Abstract

Mollusks have served as important sources of human food and medicine for a long time. Raw *Pisidium coreanum*, a freshwater bivalve of the phylum Mollusca, is used in traditional therapies in parts of Asia. However, the therapeutic effects of *Pisidium coreanum* on bone diseases are not known. We investigated the functional roles of *Pisidium coreanum* in osteoporotic bone diseases. *Pisidium coreanum* inhibited the differentiation of bone marrow-derived monocytic cells into mature osteoclasts in vitro. The ovariectomized mice that received oral administration of *Pisidium coreanum* showed improvements in both trabecular and cortical bones. This preventive activity of *Pisidium coreanum* against bone loss was due to limited osteoclast maturation with reduced osteoclast surface extent in trabecular bone tissue. The formation of large multinucleated osteoclasts in vitro was significantly decreased in response to *Pisidium coreanum*, consistent with the reduced expression levels of osteoclast markers and fusion-related genes, such as *NFATc1*, *p65*, *integrin* *αvβ3*, *DC*-*STAMP*, *OC-STAMP*, *Atp6v0d2*, *FAK*, *CD44*, and *MFR*. These data suggest that *Pisidium coreanum* inhibits osteoclast differentiation by negatively regulating the fusion of mononuclear osteoclast precursors. Thus, our data demonstrate the ability of *Pisidium coreanum* to effectively prevent estrogen-deficient osteoporosis through inhibition of multinucleated osteoclast formation.

## 1. Introduction

Mollusca, known to be the second largest phylum of invertebrate animals, encompass a variety of ecological niches. They can be classified into eight groups: Gastropoda, Bivalvia, Scaphopoda, Cephalopoda, Polyplacophora, Monoplacophora, Caudofoveata, and Solenogastres [1]. Gastropoda accounts for approximately 90% of the molluscan diversity and are found in terrestrial, freshwater, and marine habitats. The next most diverse group, the Bivalvia, is found in marine and freshwater habitats, and the other groups are found only in marine habitats. Diverse human resources, such as dyes, medicines, shells, and food, have been obtained from mollusks [1]. Natural products, including anticancer compounds, such as macrocyclic polyethers and antimicrobial peptides/proteins, were originally isolated from mollusks [2,3,4]. Mollusks are soft-bodied animals and many of them have been reported to secrete the shell, which is an external calcified rigid structure that encloses and protects their soft parts [5]. Medicinal mollusks have been traditionally consumed as folk medicine in Korea, Japan, and China. A small bivalve mollusk, *Pisidium coreanum*, 3–5 mm in size, is found in spring water near mountainous areas and has a trigonal ovate shell [6]. *P. coreanum* are reported to be ovoviviparous, with the development from embryo to post-dissoconch fry occurring in the gills [7]. A *metallothionein* gene in *P. coreanum* has been shown to encode 105 amino acids. BLAST searches and molecular phylogenetic studies of this gene revealed that *P. coreanum* is similar to freshwater bivalves, including *Dreissena polymorpha*, *Unio tumidus*, and *Crassostrea ariakensis* [8].

The bone is a key vertebrate feature that is required for the protection of soft tissues, locomotion, and mineral homeostasis [9]. It is classified into two distinct types: trabecular and cortical bone. The cortical bone (also called dense or compact bone) is mainly found in long bone shafts and consists of osteons and plexiform lamellae. Cortical bone is reported to make up about 80% of bone mass and offers strength by exhibiting resistance to bending and torsion [9,10]. Trabecular bone, which is also called cancellous or spongy bone, is primarily present in the metaphysis of long bones and in the axial skeleton. It contains a vast surface area that is generated by an interconnecting trabecular meshwork [9,11]. In adults, the rate of trabecular bone remodeling is reported to be higher than that of cortical bone remodeling [11,12].

Osteoclasts are multinucleated giant cells derived from the monocyte/macrophage hematopoietic lineage that are capable of bone resorption [13]. Two of the cytokines, macrophage colony-stimulating factor (M-CSF) and receptor activator of nuclear kappa-Β ligand (RANKL), are critical for the differentiation of osteoclast precursors into mature osteoclasts [9]. Osteoclastogenesis is a multistep process that includes several regulatory steps, including proliferation of progenitors, differentiation into mononuclear pre-osteoclasts, fusion into multinucleated osteoclasts to form the polykaryon, and activation of osteoclastic bone resorption [13]. The dynamic nature of the bone is maintained by continuous remodeling, which involves the resorption of old bone by osteoclasts and the formation of new bone by osteoblasts [9]. An imbalance in the regulation of osteoblastic bone formation and osteoclastic bone resorption in time and space can cause net bone loss and metabolic bone diseases, such as osteoporosis [14]. According to the International Osteoporosis Foundation, the global burden of osteoporosis is >200 million with 8.9 million osteoporotic fractures occurring per year. Osteoporosis can be classified as primary or secondary [15]. Primary osteoporosis can be further classified as postmenopausal or senile osteoporosis [16], and they are caused by estrogen deficiency and aging, respectively. Secondary osteoporosis is associated with other medical conditions, such as inflammation, multiple myeloma, hyperparathyroidism, Paget’s disease, and hyperthyroidism [17]. Anabolic agents (responsible for stimulating bone formation), such as parathyroid hormone and sclerostin-targeted monoclonal antibodies; and anti-catabolic agents (responsible for reducing bone resorption), such as selective estrogen receptor modulators, bisphosphonate, and anti-RANKL antibody, are critical in the management of osteoporotic bone loss [9,18]. However, owing to the side effects and low drug efficacy of the current drugs [19], further research is required to develop new drugs or formulations for the treatment of osteoporotic bone loss.

Investigating novel sources of traditional medicines is vital to the discovery of new medications for the management of osteoporosis. Raw *P. coreanum* has been traditionally used for the treatment of bone fractures. However, scientific evidence evaluating the benefits of *P. coreanum* for bone health is absent. In this study, we investigated the effects of the aqueous suspensions of *P. coreanum* powder on bone remodeling, by utilizing a postmenopausal osteoporosis animal model.

## 2. Results

### 2.1. P. Coreanum Inhibits Osteoclast Differentiation and Prevents Bone Loss in Estrogen-Deficient Mice

**Figure 1:**
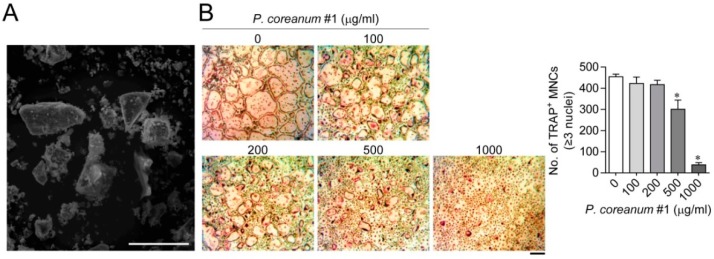
Scanning electron microscopy (SEM) images of *Pisidium coreanum* powder and the inhibitory effect of *P. coreanum* powder on osteoclast differentiation. (**A**) The dried *P. coreanum* was ground in liquid nitrogen and the surface images of the powders were obtained by SEM. Scale bar, 75 μm. (**B**) Bone marrow-derived osteoclast precursors were treated with various concentrations of *P. coreanum* #1 powder suspension in the presence of M-CSF (30 ng/mL) and RANKL (100 ng/mL) for 4 days. Cells were fixed and stained for TRAP. The number of TRAP-positive osteoclasts having more than three nuclei were counted under light microscope. * *p* < 0.01 versus control. Scale bar, 500 μm.

To produce fine powder, *P. coreanum* was dried and then ground in liquid nitrogen. The approximate average particle size from the scanning electron microscopy image was 38 μm (Figure 1A). We analyzed the effect of the aqueous suspensions of *P. coreanum* powder on the differentiation of bone marrow-derived mononuclear cells into mature osteoclasts. The powder suspension in phosphate buffered saline (PBS; pH 7.4) inhibited the formation of tartrate-resistant acid phosphatase (TRAP)-positive multinucleated cells in a dose-dependent manner (Figure 1B and Figure S1,) without affecting the cell viability (Figure S2). To investigate the effect of *P. coreanum* on bone remodeling, *P. coreanum* powder resuspended in PBS was administered orally to ovariectomized or sham-operated mice every other day for 6 weeks.

**Figure 2:**
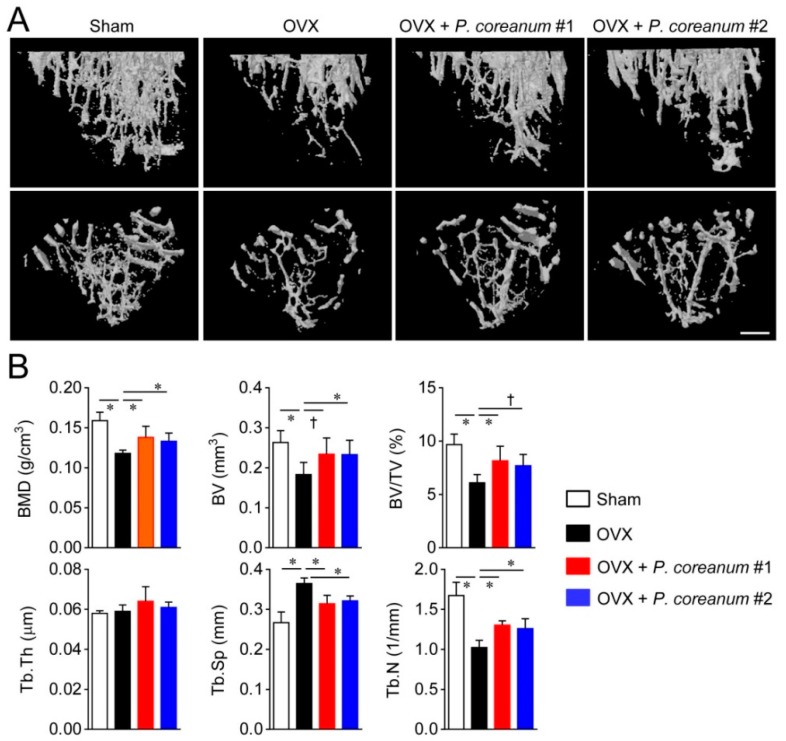
*Pisidium coreanum* powder suspension suppresses ovariectomy-induced trabecular bone loss. (**A**) Representative high-resolution micro-computed tomography (μCT) images of trabecular bone in sham-operated mice and ovariectomized mice, with or without oral administration of *P. coreanum* powder suspension (500 mg/kg) for 6 weeks. Upper panel, longitudinal section; lower panel; cross section. Scale bar, 0.5 mm. (**B**) Analyses of bone indices with the μCT data obtained from mice treated in (**A**). BMD, bone mineral density; BV, bone volume; BV/TV, BV/total volume; Tb.Th, trabecular thickness; Tb.Sp, trabecular separation; and Tb.N, trabecular number. Quantitative data are represented as means ± SDs (*n* = 7 per group). ^†^ *p* < 0.05; * *p* < 0.01.

**Figure 3:**
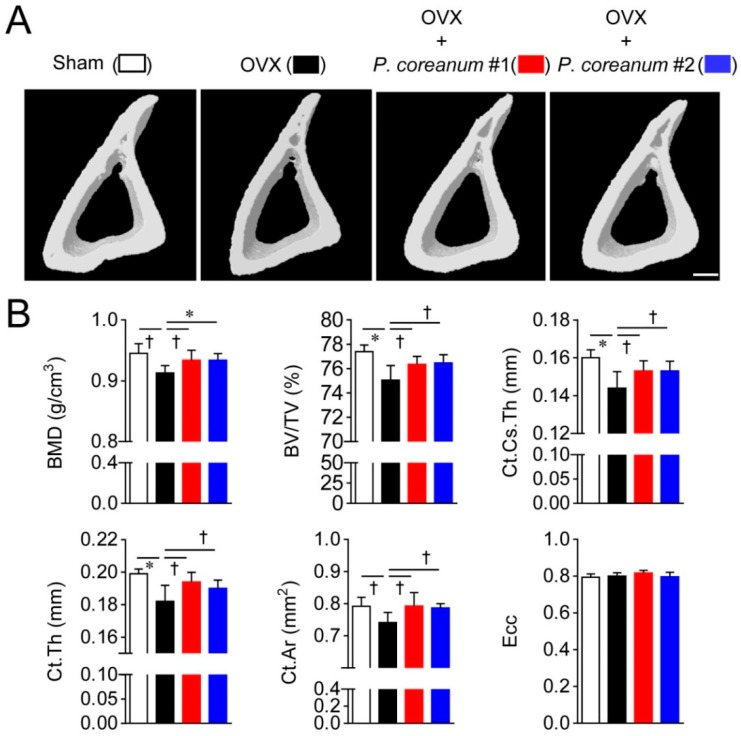
*Pisidium coreanum* powder suspension suppresses ovariectomy-induced cortical bone loss. (**A**) Representative, high-resolution, micro-computed tomography (μCT) images of tibial cortical bone in sham-operated mice and ovariectomized mice, with or without oral administration of *P. coreanum* powder suspension (500 mg/kg) for 6 weeks. Scale bar, 200 μm. (**B**) Analysis of bone indices with the μCT data obtained from mice treated in (**A**). BMD, bone mineral density; BV/TV, bone volume/total volume; Ct.Cs.Th, cortical cross-sectional thickness; Ct.Th, cortical thickness; Ct.Ar, cortical bone area; and Ecc, eccentricity. Quantitative data are represented as means ± SDs (*n* = 7 per group). ^†^ *p* < 0.05; * *p* < 0.01.

We assessed the structural changes in the microarchitecture of the trabecular and cortical bone in response to the powdered clam. Trabecular bone microstructure analyses of long bones by high-resolution micro-computed tomography (μCT) showed that the ovariectomized mice exhibited an osteoporotic phenotype (Figure 2). *P. coreanum* powder suspension from two locations in Korea ameliorated the osteoporotic characteristics associated with estrogen deficiency, resulting in elevated bone mineral density (BMD), bone volume/ total volume (BV/TV), trabecular thickness (Tb.Th), and trabecular number (Tb.N), and decreased trabecular separation (Tb.Sp) compared to the levels in the ovariectomized mice (Figure 2). We also analyzed the effect of *P. coreanum* powder suspension on cortical bone morphometric parameters. *P. coreanum* powder suspension induced elevations in cortical bone BMD and BV/TV, cortical cross-sectional thickness (Ct.Cs.Th), cortical bone area (Ct.Ar), and cortical thickness (Ct.Th) (Figure 3). Collectively, our data shows that *P. coreanum* powder suspension plays a positive role in suppressing ovariectomy-induced osteoporotic bone loss by improving trabecular and cortical bone.

### 2.2. P. Coreanum Inhibits the Fusion during Osteoclast Maturation

**Figure 4:**
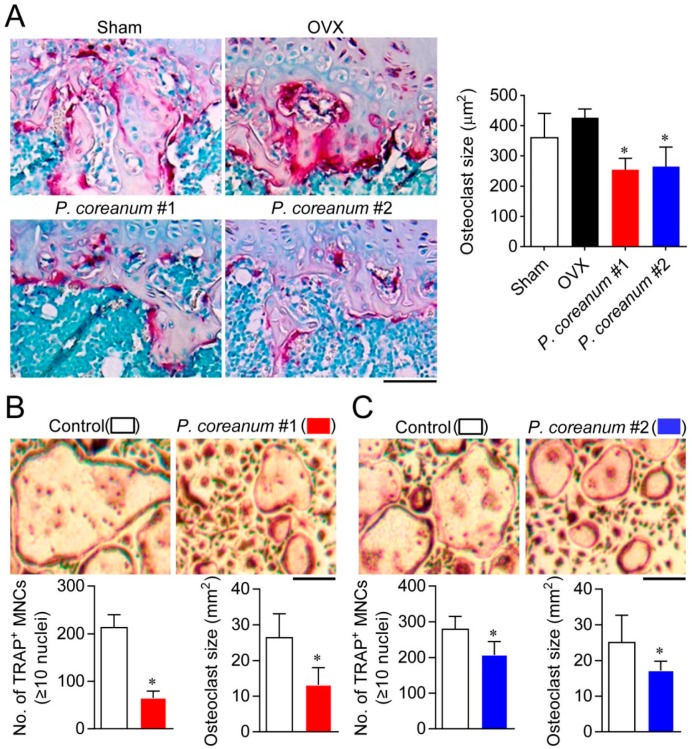
The decreased size and number of multinucleated osteoclasts by treatment with *Pisidium coreanum* powder suspension. (**A**) Histomorphometric analysis of the trabecular osteoclasts. Representative high-magnification images of TRAP-positive osteoclasts on trabecular bone surface in sham-operated mice and ovariectomized mice, with or without oral administration of *P. coreanum* powder suspension (500 mg/kg) for 6 weeks. * *p* < 0.01 versus OVX. Scale bar, 200 μm. (**B**,**C**) The number and size of TRAP-positive multinucleated osteoclasts containing more than ten nuclei was determined after treatment of bone marrow-derived osteoclast precursors with *P. coreanum* powder suspension (numbers 1 and 2) in the presence of M-CSF and RANKL. Multinucleated osteoclast size was analyzed in 90 multinucleated cells from four or more random fields using ImageJ software. * *p* < 0.01 versus control. Scale bar, 500 μm.

Next, we analyzed the effect of *P. coreanum* on osteoclast size both in vivo and in vitro. The size of multinucleated osteoclasts on trabecular bone surfaces was decreased in the ovariectomized mice administrated with the powder suspension of *P. coreanum* compared to the control-treated ovariectomized mice (Figure 4A). Additionally, the powder suspension suppressed the formation of large multinucleated osteoclasts containing more than 10 nuclei, showing a significant decrease in osteoclast size compared with the control-treated osteoclasts (Figure 4B). These results suggest that the powder suspension of *P. coreanum* inhibits osteoclast multinucleation process.

**Figure 5:**
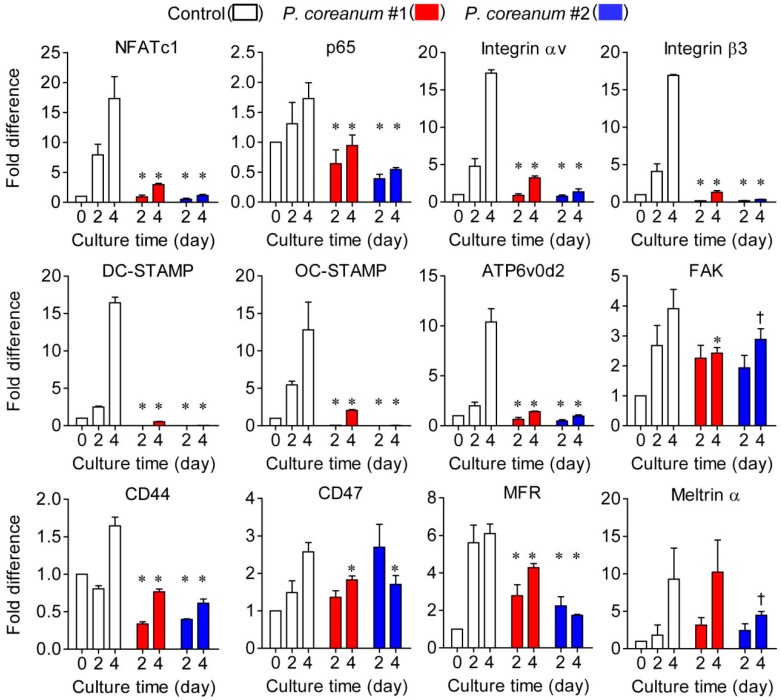
*P. coreanum* powder suspension negatively regulates the mRNA expression levels of osteoclastogenic and fusion-related genes. Expression levels of osteoclast markers and fusion-related genes, including *NFATc1*, *p65*, *integrin αv*, *integrin β3*, *DC-STAMP*, *OC-STAMP*, *ATP6v0d2*, *FAK*, *CD44*, *CD47*, *MFR*, and *meltrin-α*, during osteoclast differentiation, were measured using quantitative real-time PCR. Data are means ± SDs (*n* = 3). ^†^ *p* < 0.05 versus control; * *p* < 0.01 versus control.

Gene expression levels of NFATc1, a master transcription factor for osteoclast differentiation [20]; p65; and integrin αvβ3, which plays an important role in regulating cell adhesion, migration, differentiation, and bone resorption in osteoclasts [21], were markedly inhibited in response to administering *P. coreanum* powder suspension (Figure 5). Additionally, it should be noted that the expression levels of osteoclast fusion-related genes such as *DC-STAMP*, *OC-STAMP*, *Atp6v0d2*, *FAK*, *CD44*, and *MFR* were significantly reduced by the treatment with *P. coreanum* powder suspension (Figure 5). These results suggest that *P. coreanum* inhibits osteoclast differentiation by negatively regulating the fusion stage of osteoclast maturation process.

## 3. Discussion

Whole-body powders, ground shells, and mother of pearl extracts from bivalve mollusks, such as oysters, pearl oysters, clams, and mussels, have long been used in conventional and traditional medicine [1]. Studies have shown that oyster shell electrolysate significantly increases bone mineral density in osteoporosis patients and is used in the homeopathic treatment of bone deficiencies [1,22]. Bioactive lipids from mussels containing polyunsaturated fatty acids have been effective in preventing and treating rheumatoid arthritis [23]. Gastrointestinal digests of the intestines isolated from abalone have been shown to induce osteoblast differentiation by promoting bone morphogenetic protein 2 (BMP2) expression and stimulating alkaline phosphatase activity [24]. Interestingly, the 16 expressed sequence tags from *P. coreanum* have been shown to exhibit high similarities to BMP2 [25], suggesting that this freshwater clam species could provide potential regulators of bone cells and promoters of bone health.

The mononuclear osteoclast precursors fuse to form multinucleated giant osteoclasts, representing bone-resorbing function [26]. In the present study, we showed that osteoclast size on trabecular bone surfaces was markedly reduced in the ovariectomized mice administered with the powder suspension of *P. coreanum* compared to the untreated control ovariectomized mice. When treated with the powder suspension of *P. coreanum* during osteoclast differentiation, there was a substantial reduction in the number of TRAP-positive osteoclasts containing more than 3 or 10 nuclei and in osteoclast size. Osteoclast size heterogeneity has been reported to be associated with differences in bone resorptive activity. Large osteoclasts were found to resorb a greater total area of bone slices per cell than small osteoclasts [27]. Small osteoclasts were shown to express lower mRNA levels of MMP-9, cathepsins K, and cathepsins L when compared to large osteoclasts [28]. Therefore, the inhibitory effect of *P. coreanum* on multinucleated osteoclast formation in vitro and estrogen-deficient osteoporosis in vivo can be explained, at least in part, by the regulation of osteoclast size. Consistently, several osteoclast fusion-related genes, including *DC-STAMP*, *OC-STAMP*, *Atp6v0d2*, *FAK*, and *MFR* were downregulated in response to treatment with *P. coreanum* powder suspension (Figure 5). DC-STAMP has been reported to be critical for cell-cell fusion in osteoclasts [29]. Multinucleation was completely abrogated in DC-STAMP-defected osteoclasts and DC-STAMP-deficient mice, and they showed increased bone mass with the decreased bone-resorption activity. Atp6v0d2 null mice showed osteoporotic symptoms due to impaired osteoclast fusion and increased bone formation [30]. OC-STAMP, MFR, and FAK were also reported to promote the formation of multinucleated osteoclasts [31,32,33]. Our data strongly suggest that *P. coreanum* powder affects the fusion and multinucleation process during osteoclast differentiation.

Nacre is one of the strongest known shell layers and has been studied from the perspective of a biomaterial. Previous studies on the effects of molluscan-derived materials on bone metabolism were conducted predominantly using nacre. [34]. It is a composite material composed of inorganic calcium carbonate plates, which are connected by a complex organic matrix, constituting the inner layer of pearl oysters and shells of freshwater pearl mussel [35]. Previous in vitro and in vivo studies, including ours, have shown that nacre obtained from marine bivalves (including pearl oysters) majorly contribute to regulating bone remodeling by inhibiting osteoclast function and stimulating osteoblast function [36,37,38,39]. Nacre also shows excellent biocompatibility with bone tissue and biodegradability [40,41].

In conclusion, our study showed that *P. coreanum* powder suppresses osteoclast differentiation by inhibiting the fusion of mononuclear osteoclast precursors, and thus helps to maintain trabecular and cortical bone in an estrogen deficiency-induced osteoporosis animal model. To the best of our knowledge, this is the pioneer scientific study describing the effects of *P. coreanum* on bone metabolism and osteoporosis. Further characterization of anti-osteoporotic activities of the soft body, shell, and bioactive metabolites (proteins, peptides, lipids, and carbohydrates) of *P. coreanum* could reveal the full therapeutic effects of *P. coreanum* on osteoporosis.

## 4. Materials and Methods

### 4.1. Preparation of P. coreanum Powder

*P. coreanum* samples were obtained from two different farms (*P. coreanum* numbers 1 and 2) in Danyang-gun and Chungcheongbuk-do, Korea. The samples were washed three times using deionized distilled water and were dried at 60 °C in a drying oven for 3 days. Dried *P. coreanum* was ground in liquid nitrogen using a mortar and pestle. The resulting powders were dried at 60 °C in a drying oven overnight and were resuspended in PBS (pH 7.4). After vigorous vortexing for 30 min, the aqueous suspensions of *P. coreanum* powder were incubated for 12 h at 4 °C with rocking and were filtered using strong nylon mesh with 40 µm pore size (Corning Incorporated, Durham, NC, USA). Immediately after mixing thoroughly, each time by vigorous vortexing for 30 s, the suspensions were used to treat mice or bone marrow-derived mononuclear osteoclast precursors.

### 4.2. Scanning Electron Microscopy Analysis

The surface morphology of the *P. coreanum* powdered samples was evaluated using scanning electron microscopy. The specimens were fixed using 2.5% glutaraldehyde in PBS for 2 h, washed three times with PBS, and fixed with 2% OsO_4_. The samples were then washed three times with PBS and dehydrated by sequential washes in 70%, 80%, 90%, 95%, and 100% ethanol. They were dried at critical-point, mounted on aluminum stubs with conductive silver paint, and then coated with Pt–Pd using a Hitachi E-102 ion sputter from Hitachi (Fukuoka, Japan). The *P. coreanum* powdered samples were observed under the S-2500 scanning electron microscope from Hitachi (Japan), at 25 kV.

### 4.3. Osteoclast Differentiation

Bone marrow-derived mononuclear osteoclast precursors were isolated from tibiae and femurs of 6-week-old male C57BL/6J mice (Central Lab Animal, Seoul, Korea) by flushing the bone marrow cavity. Cells were treated with red blood cell lysis buffer (Sigma-Aldrich, St. Louis, MO, USA), and then incubated with minimum essential medium-alpha (α-MEM; Hyclone, Logan, UT, USA) supplemented with 10% fetal bovine serum (FBS; Hyclone) and M-CSF (5 ng/mL) for 12 h. The non-adherent cells were harvested and were further cultured for 3 days with α-MEM containing M-CSF (30 ng/mL) to generate osteoclast precursors. Osteoclast differentiation was induced by incubating osteoclast precursors in the presence of M-CSF (30 ng/mL) and RANKL (100 ng/mL) with or without the aqueous suspensions of *P. coreanum* powder for 4 days. TRAP staining was performed with the use of a leukocyte acid phosphatase staining kit (Sigma-Aldrich), following the manufacturer’s instructions. TRAP-positive multinucleated cells having more than three or ten nuclei were counted using a light microscope. For measuring the size of TRAP-positive multinucleated osteoclasts containing more than ten nuclei, 90 multinucleated cells from four or more random fields were traced using ImageJ software (NIH, Bethesda, MD, USA).

### 4.4. MTT Assay

Osteoclast precursors were seeded on a 48 well culture plate in the presence of M-CSF (30 ng/mL) and were treated with or without *P. coreanum* powder (500 μg/mL) for 24 h. A 3-(4,5-dimethylthiazol-2-yl)-2,5-diphenyltetrazolium bromide (MTT) solution (0.5 mg/mL) was added, and that was followed by 1 h incubation. The absorbance was determined at 570 nm using a 96 well microplate reader (BioRad, Hercules, CA, USA).

### 4.5. Ovariectomy

Seven-week-old female C57BL/6 mice were purchased from SAMTAKO Bio Korea Co., Ltd. (Osan, Korea) and were housed in the laboratory animal facility of Yeungnam University College of Medicine. Mice were provided with a standard rodent chow diet and water ad libitum. They were maintained under 12 h light/12 h dark illumination cycles, at 20–25 °C and 60% relative humidity. The mice were allowed to acclimatize for a week before commencing experiments. All animal experiments were approved by the Institutional Animal Care and Use Committee of Yeungnam University College of Medicine and were in compliance with the Guide for the Care and Use of Laboratory Animals. For ovariectomy experiments, 8 week old female C57BL/6 mice were anesthetized by intraperitoneal injection of 2.5% avertin and were subjected to sham surgery or bilateral ovariectomy to induce osteoporosis, as described previously [30]. *P. coreanum* powdered samples were suspended in phosphate buffered saline (PBS) (pH 7.4) and orally administered to mice every 2 days for 6 weeks, beginning 3 days post-surgery. The mice were sacrificed for analysis after 6 weeks.

### 4.6. High-Resolution μCT Analysis

The bilateral tibiae were fixed in 3.7% formaldehyde solution for preservation and scanned by high-resolution μCT using SkyScan 1272 from SkyScan (Kontich, Belgium), with an 8 μm pixel size, 166 kV source voltage, 60 μA current, and 0.25 mm aluminum filter. The whole bone images were reconstructed by the SkyScan software (version 1.4.4) and their lengths were measured. The regions analyzed were approximately 1.7 mm distal to the growth plate in the trabecular bone and a proximal/middle site (37% of the bone’s length from its proximal end) in the cortical bone of the tibiae. Three-dimensional structural analyses for these trabecular and cortical bone sites were performed using the SkyScan software (version 1.6.1.1). We evaluated the bone mineral density (BMD; g/cm^3^), bone volume (BV; mm^3^), bone volume/ total volume (BV/TV; %), trabecular thickness (Tb.Th; mm), trabecular separation (Tb.Sp; mm), and trabecular number (Tb.N; 1/mm) in the trabecular bone; and bone mineral density (BMD; g/cm^3^), cortical bone area/ total area (Ct.Ar/Tt.Ar; %), cortical cross-sectional thickness (Cs.Th; mm), cortical thickness (Ct.Th; mm), cortical bone area (Ct.Ar; mm^2^), and eccentricity (Ecc) in the cortical bones.

### 4.7. Quantitative Real-Time RT-PCR

Total RNA was isolated from the cells using the Trizol reagent (Invitrogen, Carlsbad, CA, USA); 2 μg of the RNA was subjected to reverse transcription for 1 h at 42 °C with oligo(dT) using an M-MLV RT kit (Invitrogen). Real-time PCR was carried out using LightCycler 480 SYBR Green I Master with LightCycler 96 Real-Time PCR (Roche, Mannheim, Germany), and the data were analyzed using Light Cycler software (Roche). Normalized mRNA abundance was determined with the comparative delta threshold cycle method, and GAPDH mRNA was used as an internal reference control. The sequences of PCR primers are listed in Table S1.

### 4.8. Statistical Analyses

Quantitative data are presented as means ± standard deviations (SD) from the indicated number of mice, and the data generated from more than three groups were assessed using analysis of variance (ANOVA), utilizing the SPSS 18.0 software package (SPSS Inc., Chicago, IL, USA). A *p*-value < 0.05 was considered statistically significant.

## Supplementary Materials

Supplementary materials can be found at https://www.mdpi.com/1422-0067/20/23/6076/s1.
