# Supplementary material for: Pisidium coreanum Inhibits Multinucleated Osteoclast Formation and Prevents Estrogen-Deficient Osteoporosis
Source: Int J Mol Sci. 2019 Dec 2;20(23):6076. doi: 10.3390/ijms20236076 (PMC6929078; doi:10.3390/ijms20236076)
Supplement: Supplementary file 1 [file ijms-20-06076-s001.pdf]

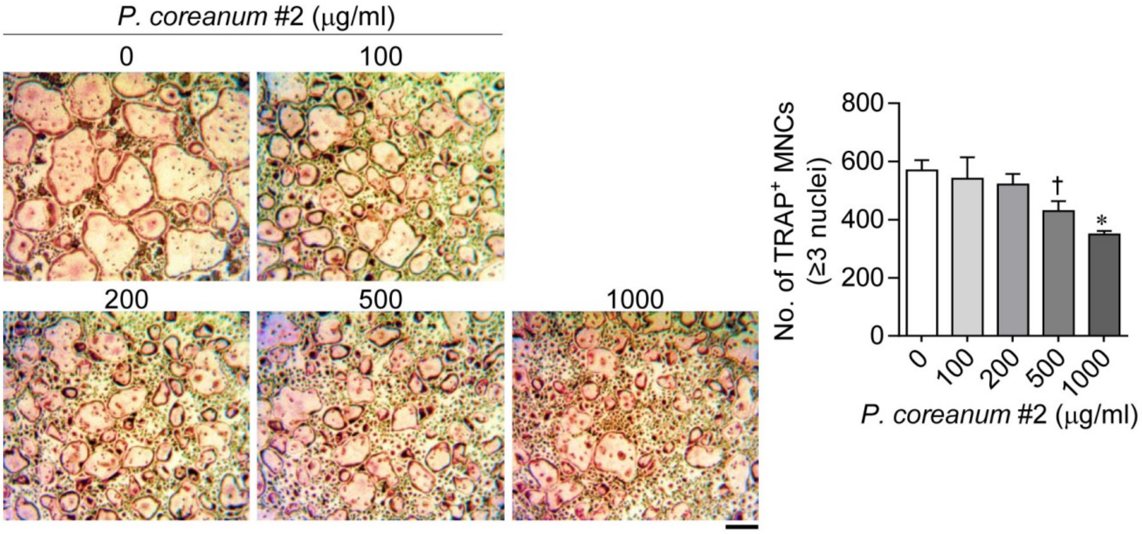

Figure S1. Inhibitory effect of *P. coreanum* powder suspension on osteoclast differentiation. Bone marrow-derived osteoclast precursors were treated with various concentrations of *P. coreanum* #2 powder suspension in the presence of M-CSF (30 ng/mL) and RANKL (100 ng/mL) for 4 days. Cells were fixed and stained for TRAP. The number of TRAP-positive osteoclasts having more than three nuclei was counted under a light microscope. †$p < 0.05$; *$p < 0.01$. Scale bar, 500 μm.

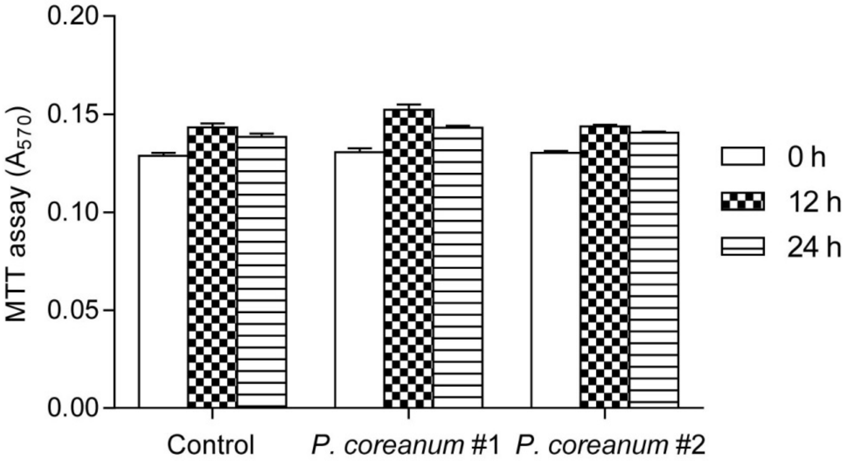

Figure S2. Cytotoxic effect of *P. coreanum* powder suspension on osteoclast precursors. Osteoclast precursors were incubated with *P. coreanum* powder suspension (500 μg/mL) for 12 h or 24 h and cell viability was assessed using MTT assay. Each value represents the mean of three independent experiments ± S.D ($n = 3$).

Table S1. Sequences of PCR primers used in this study.

**Gene**	**Sense (5′→3′)**	**Antisense (5′→3′)**
ATP6v0d2	TCAGATCTCTTCAAGGCTGTGCTG	GTGCCAAATGAGTTCAGAGTGATG
DC-STAMP	AGACGTGGTTTAGGAATGCAG	GGCTGGAAGTTCACTTGAAAC
OC-STAMP	CCTTGGTGCTACAGGCCTAC	CAGAGTCCGAGTTCCCTGTC
Meltrin-α	AAATCCCACGACAATGCTCAGC	CCAGCTCATGTGCCAAGGTCA
CD44	GGACTCCAGGGGGAGTTCCCGCAC	CGTCCCATTGCCACCGTTGATCAC
MFR	AAATCAGTGTCTGTTGCTGCTGG	CTGGGGTGACATTACTGATAC
αv	CCTCAGAGAGGGAGATGTTCACAC	AACTGCCAAGATGATCACCCACAC
β3	GATGACATCGAGCAGGTGAAAGAG	CCGGTCATGGATAGTGATGAGTAG
CD47	AGGAGGAGAAAGGAGGTTGC	AACCACGATGACTGTGAGCA
FAK	GGCAGCTGCTTATCTTGACC	TGATGCCCCTGACATCAGTA
NFATc1	GAGACAGACATCGGGAGGAAGA	GTGGGATGTGAACTCGGAAGA
p65	GCGTACACATTCTGGGGAGT	GTTAATGCTCCTGCGAAAGC
Gapdh	AGGTCGGTGTGAACGGATTTG	TGTAGACCATGTAGTTGAGGT
